# CARI III Inhibits Tumor Growth in a Melanoma-Bearing Mouse Model through Induction of G0/G1 Cell Cycle Arrest

**DOI:** 10.3390/molecules190914383

**Published:** 2014-09-12

**Authors:** Hye-Jin Park

**Affiliations:** Department of Food Science and Biotechnology, College of BioNano Technology Gachon University, Sungnam Gyeonggi-do 461-701, Korea; E-Mail: nimpi79@hanmail.net; Tel.: +82-31-750-5382; Fax: +82-2-455-5517

**Keywords:** mushroom mixture, CARI III, melanoma, G0/G1 cell cycle arrest, anti-cancer, cyclin D1, CDK4, CDK2

## Abstract

Mushroom-derived natural products have been used to prevent or treat cancer for millennia. In this study, we evaluated the anticancer effects of CARI (Cell Activation Research Institute) III, which consists of a blend of mushroom mycelia from *Phellinus linteus* grown on germinated brown rice, *Inonotus obliquus* grown on germinated brown rice, *Antrodia camphorata* grown on germinated brown rice and *Ganoderma lucidum*. Here, we showed that CARI III exerted anti-cancer activity, which is comparable to Dox against melanoma *in vivo*. B16F10 cells were intraperitoneally injected into C57BL6 mice to develop solid intra-abdominal tumors. Three hundred milligrams of the CARI III/kg/day p.o. regimen reduced tumor weight, comparable to the doxorubicin (Dox)-treated group. An increase in life span (ILS% = 50.88%) was observed in the CARI III-administered group, compared to the tumor control group. CARI III demonstrates anti-proliferative activity against B16F10 melanoma cells through inducing G0/G1 cell cycle arrest. CARI III inhibits the expression of cyclin D1, CDK4 and CDK2 and induces p21. Therefore, CARI III could be a potential chemopreventive supplement to melanoma patients.

## 1. Introduction

Malignant melanoma is one of the most aggressive skin cancers with a high metastatic potential and extraordinary resistance to anti-cancer agents [1]. Currently, 2–3 million non-melanoma and 132,000 melanoma skin cancers occur globally each year [2]. If detected early and surgically excised, the five-year survival rate is satisfactory; however, late-stage cancer is difficult to treat, and long-term survival is low. Despite partial success gained by the use of platinum analogues, nitrosoureas, taxanes, Vinca alkaloids and cytokines [3], presently there is no effective chemotherapy against malignant melanoma. Among the drugs exerting anti-cancer activity against malignant melanoma, dacarbazine has been extensively used, though it could achieve only a response rate of 11%–25% and a short survival time [4]. Therefore, it is necessary to develop anti-cancer agents with potent activity and weak side effects against melanoma.

Medicinal mushrooms have been used to treat cancer for thousands of years. Unlike Western medicine, which generally uses pure compounds targeting a single molecule or pathway, these mushrooms contain multiple components that interact with multiple molecular targets [5]. Recently, a number of bioactive molecules, including anti-tumor agents, have been originated from various mushrooms. There is much anecdotal evidence of the efficacy of mushroom mixtures in cancer patients. However, systematic preclinical evaluation of these mushrooms is necessary. Recently, mushroom mixtures were found to have even stronger activities when using mushroom alone [6]. 

*Phellinus linteus* (*P. linteus*) belongs to the Hymenochaetaceae basidiomycetes, and it is commonly called “Sanghwang” in Korean. *P. linteus* is a well-established medicinal mushroom that has been used to treat various human cancers in Asia [7]. We have recently shown that *Phellinus linteus* grown on germinated brown rice (PBR) has anti-tumor activities, which induce apoptosis associated with cell cycle arrest at the G0/G1 phase in human colon cancer cells [8].

*Inonotus obliquus* (IO), commonly known as the chaga mushroom, belongs to the *Inonotus* genus and the Hymenochaetaceae family of basidiomycetes. IO is found in the birch forests of Russia, Korea, Eastern and Northern Europe, northern areas and the North Carolina Mountains of the USA and Canada. There have been numerous studies demonstrating its antioxidant, anti-tumor and antimicrobial activities [9].

*Antrodia camphorata* (AC) is an indigenous and rare mushroom of the Polyporaceae family that only grows on the inner heart rot of the native Taiwanese tree, *Cinnamomum kanehirai* Hay (Lauraceae). AC is used in traditional Chinese medicine for treating food and drug poisoning, diarrhea, abdominal pain, hypertension, pruritus (skin itch) and liver dysfunction [10]. Our group reported that the ethanol extract of AC on germinated brown rice (CBR) suppresses inflammatory responses in mice with acute DSS (Dextran Sodium Sulfate)-induced colitis [11]. AC grown on germinated brown rice exerted inhibitory activity against HT-29 human colon carcinoma proliferation through inducing G0/G1 phase arrest and apoptosis by targeting the beta-catenin signaling [11], as well as suppressed melanoma cell proliferation by inducing apoptosis and cell differentiation and tumor growth [12].

*Ganoderma lucidum* ((Leyss. ex Fr.) Karst), a popular medicinal mushroom, is a basidiomycete belonging to the Polyporaceae. For hundreds of years, this mushroom has been regarded as a folk medicine used for the prevention and treatment of various human diseases, such as hepatitis, hypertension, chronic bronchitis, bronchial asthma, cancer and others in China [13]. Numerous studies have described that the major bioactive components in *G. lucidum* are polysaccharides, ganoderic acid (triterpene) and adenosine [14].

We evaluated various formulations of mushroom mixtures to choose the most effective one against B16F10 mouse melanoma cells, both *in vitro* and *in vivo*. Based on preliminary results, we decided to further evaluate the effects of CARI (Cell Activation Research Institute) III, a mushroom mixture, composed of *Phellinus linteus* grown on germinated brown rice, *Inonotus obliquus* grown on germinated brown rice, *Antrodia camphorata* grown on germinated brown rice and *Ganoderma lucidum*.

In the present study, we evaluated the effects of mushroom mixture CARI III on the growth and invasive behavior of B16F10 melanoma cells *in vitro* and *in vivo*.

## 2. Results and Discussion

**Figure 1:**
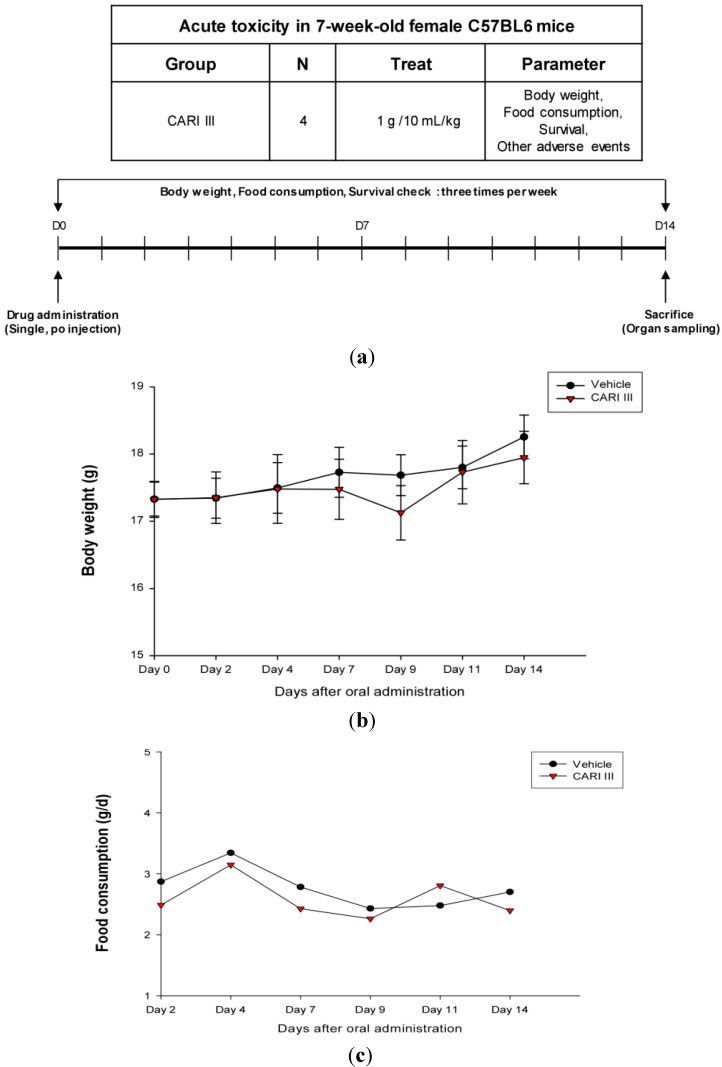
The effect of CARI (Cell Activation Research Institute) III on body weight and food consumption in the acute toxicity study. (**a**) Scheme of the acute toxicity study. (**b**) Body weight is presented as the mean ± SE (bars). Average diet consumption per mouse per day is presented once a day. The two groups represented below are the control group and the CARI III group (1000 mg/kg). (**c**) Daily food intake consumption.

The body weight and general appearance of each mouse were measured and observed every day as evidence of systemic toxicity. In the acute toxicity study, doses up to 1,000 mg/kg did not exhibit any mortality or any signs of toxicity (e.g., changes in body weight and the amount of food consumption) after oral administration of a single dose (Figure 1). This dose may be considered as the no observed adverse effect level (NOAEL) [15] for CARI III. Furthermore, the toxicity guideline [16] discourages studies beyond 1 g/kg if no abnormality is observed.

**Figure 2:**
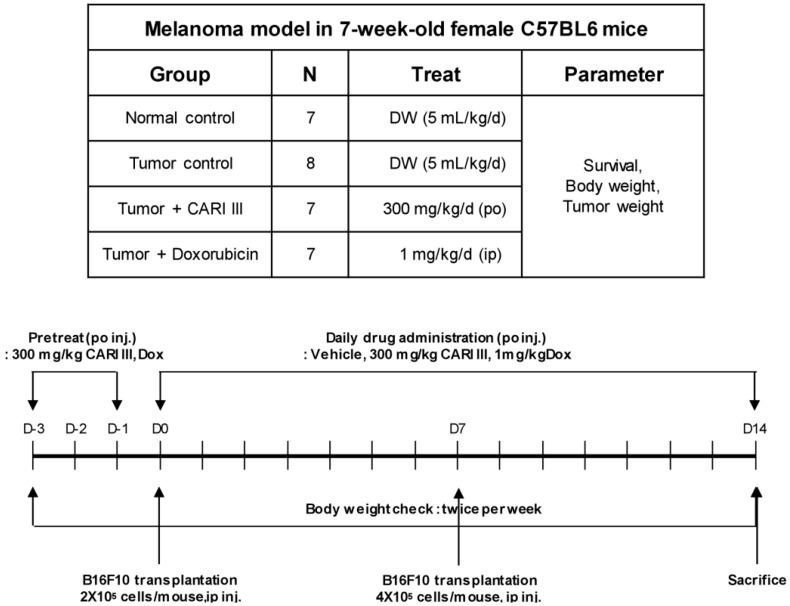
Experimental design of the effect of CARI III or doxorubicin (Dox) on B16F10 melanoma xenograft in an animal model.

**Figure 3:**
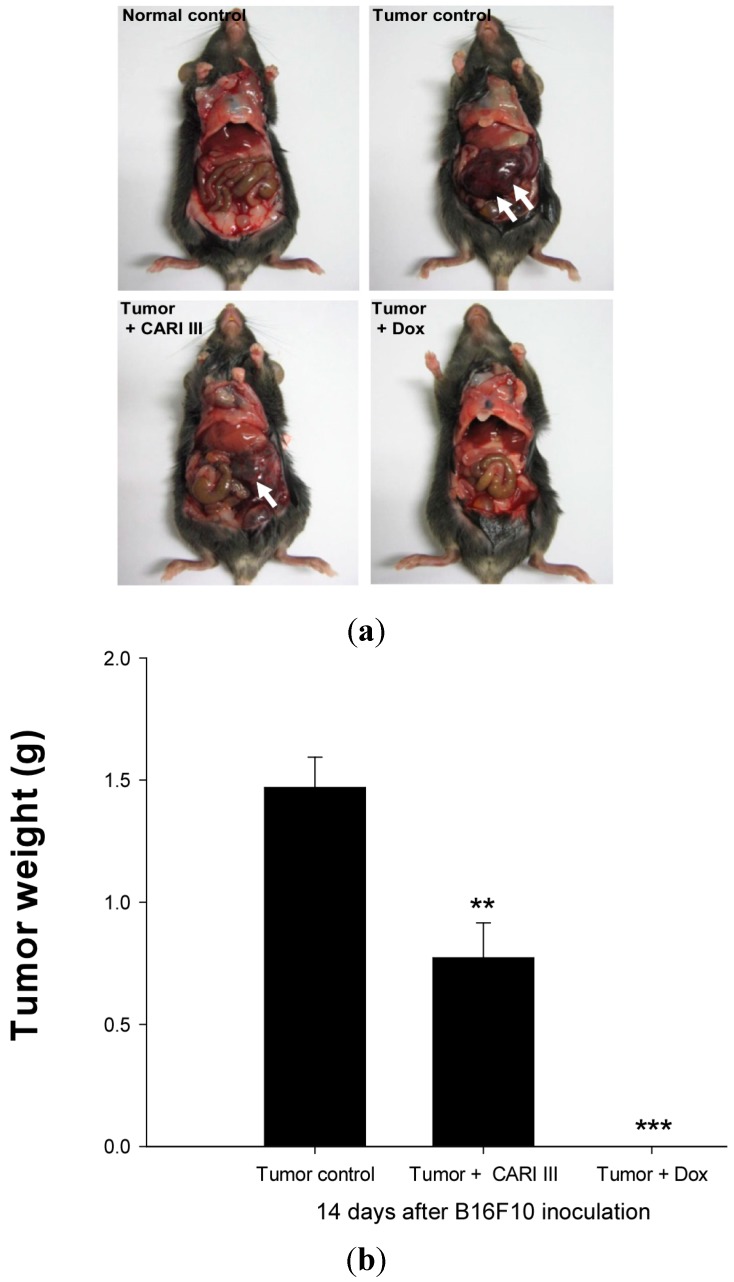
The effect of CARI III on tumor growth in B16F10 melanoma-bearing mice. (**a**) Representative mice were administered with CARI III, doxorubicin (1 mg/kg/day) or DW (distilled water). Mice were sacrificed on Day 14 after B16F10 inoculation. (**b**) Tumor weight changes on B16F10-inoculated mice treated with DW, CARI III (300 mg/kg/day) and Dox (1 mg/kg/day). Data were obtained from the three independent experiments and were expressed as the means ± SE. One-way ANOVA was used for the comparison of multiple group means, followed by Dunnett’s *t*-test (** *p* < 0.01, *** *p* < 0.001 *vs*. Tumor control).

We examined whether CARI III could suppress tumor growth *in vivo*. Intraperitoneal injection of B16F10 into C57BL6 mice developed into solid intra-abdominal tumors. Either CARI III or saline was orally administered three days before tumor cell implantation (Figure 2). The mean tumor weights in CARI III-treated mice (300 mg/kg/day) were significantly inhibited compared with those of the tumor control group. Doxorubicin (Dox) (1 mg/kg i.p., daily, for 17 days), used as the positive control, also inhibited tumor growth and tumor weight significantly (*p* < 0.001 and *p* < 0.001, Figure 3). Doxorubicin (Dox) is one of the most widely used antitumor drugs against a lot of solid tumors, including melanoma. Although Dox had been shown to exert robust antitumor activity, its effectiveness was often restricted by drug-resistance and dose-dependent side effects, especially Dox-induced cardiomyopathy [17]. This study showed that the 300 mg of CARI III/kg/day regimen reduced tumor weight, comparable to the doxorubicin (Dox)-treated group.

**Figure 4:**
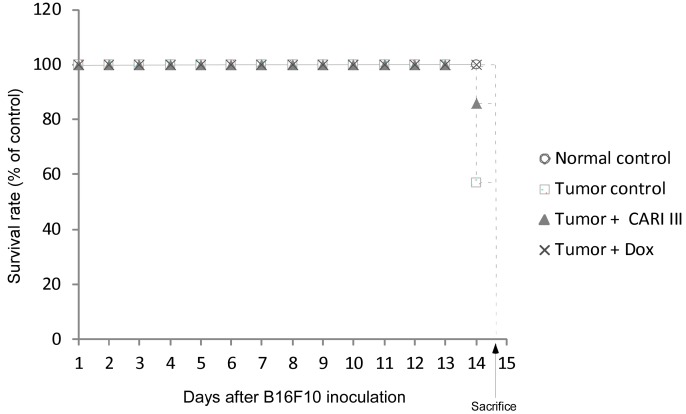
CARI III increases the survival rate of B16F10 melanoma-bearing mice. Kaplan–Meier curves for B16F10 murine neoplasms in C57BL/6 mice. The number of animals per group was seven (*n* = 7).

The effects of CARI III on the survival rate of mice are shown in Table 1. The survival rate of mice treated with CARI III was prolonged compared to that of the tumor control group. Survival rates were 57% and 86%, for the DW (distilled water)- and CARI III-treated group, respectively. A Kaplan–Meier plot illustrates that the survival rate of CARI III extract-treated mice was increased when compared to tumor control animals. The maximum survival rate was observed in the CARI III-administered group, as shown in Figure 4. The percentage increase in life span = T − C / C × 100, where T and C are the percent of surviving mice in the treated and tumor control groups. An increase in life span (ILS% = 50.88%) was obtained in the CARI III-administered group, compared to the tumor control group.

**Table 1:** CARI III increases the survival rate of B16F10 melanoma-bearing mice.

Group	Initial	Final	Death	Survival Rate (%) at Day 14
**Normal control**	6	6	0	100
**Tumor control**	6	4	3	57
**Tumor + CARI III**	7	6	1	86
**Tumor + Dox**	7	7	0	100

**Figure 5:**
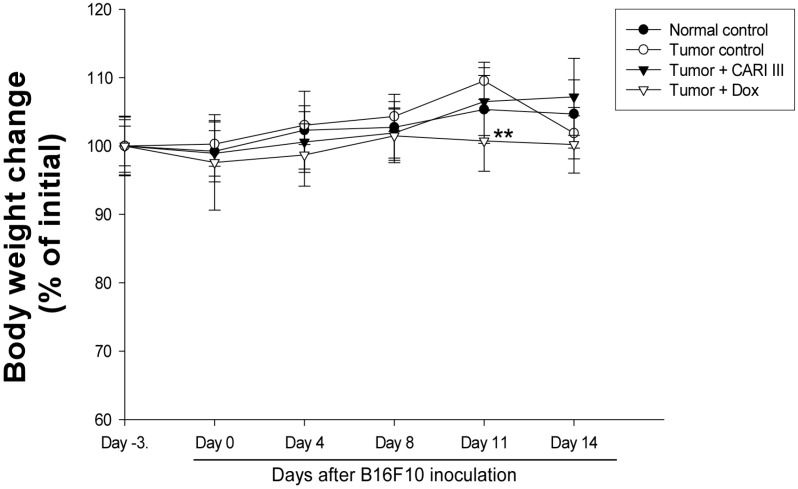
Effect of CARI III on body weight. The body weight of the mice was measured during treatment. The mean body weights before CARI III treatment in each group are expressed as 100%. The body weight of the following days is expressed as the ratio of the body weight to the initial body weight. Data were obtained from the three independent experiments and were expressed as the means ± SE. One-way ANOVA was used for the comparison of multiple group means, followed by Dunnett’s *t*-test (** *p* < 0.01 *vs*. Tumor control).

The body weights of the CARI III-treated mice were similar to those of the normal control group (Figure 5), indicating that there were no significant side effects during treatments. Data indicate that melanoma growth was significantly suppressed in mice treated with CARI III without loss of body weight, while the Dox-treated mice showed significant body weight loss. Based on the observation of body weight (Figure 5), a small amount of host toxicities were observed after Dox treatment.

**Figure 6:**
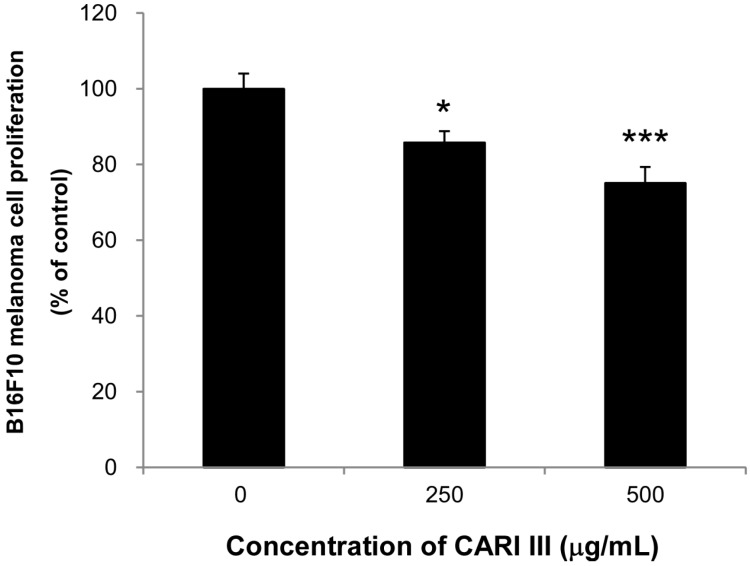
Anti-proliferative effects of CARI III extracts against B16F10 melanoma. Cells were treated with the indicated concentrations of CARI III extracts for 72 h. Cell viability was compared to the control, and the statistically different levels are denoted by: * *p* < 0.05, *** *p* < 0.001. Data were obtained from the three independent experiments and are expressed as the means ± SE.

Next, we evaluated if the mixture of mushrooms also could inhibit the growth of highly invasive B16F10 cancer cells *in vitro*. As seen in Figure 6, CARI III markedly suppressed proliferation of B16F10 cells at 250 and 500 μg/mL (Figure 6) (*p* < 0.05 *vs*. control).

**Figure 7:**
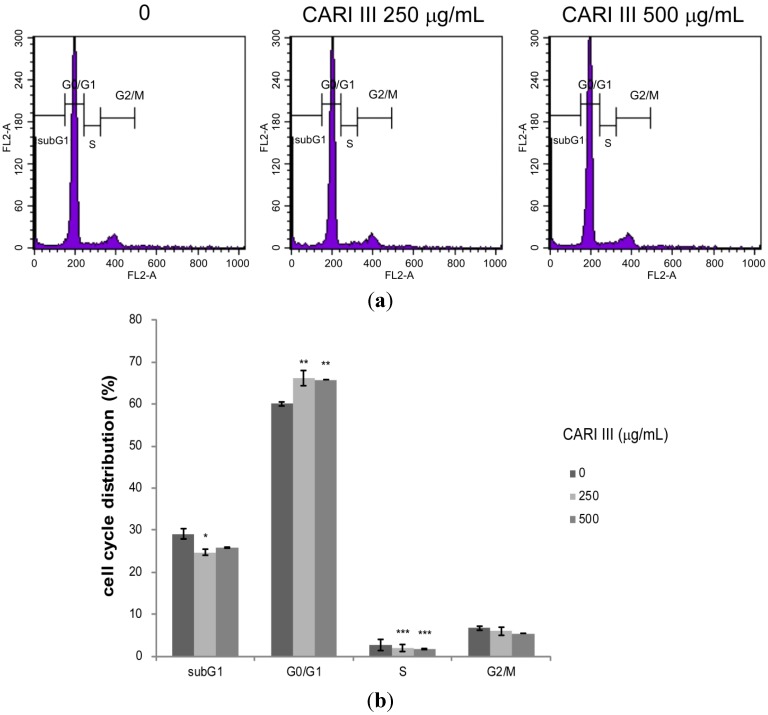
The effect of CARI III extract on the B16F10 melanoma cell cycle. Cells were analyzed by flow cytometry. (**a**) Histograms represent the findings during sub-G1, G0/G1, S and G2/M phases of B16F10 cells. (**b**) Quantitative analysis of the cell cycle distribution. Data were obtained from the three independent experiments and were expressed as the means ± SE. One-way ANOVA was used for the comparison of multiple group means, followed by Dunnett’s *t*-test (* *p* < 0.05, ** *p* < 0.01, *** *p* < 0.001 *vs*. control).

Deregulation of the cell cycle machinery has been associated with cancer initiation and progression [12]. To elucidate the mechanism of cell growth inhibition by the CARI III mushroom complex, we determined its effect on cell cycle distribution. Exposure of B16F10 cells to the CARI III mushroom complex resulted in the enrichment of the G0-G1 fraction, which was accompanied by a decrease in the S phase (Figure 7).

The rates of the G0-G1 population in CARI III-treated cells were 60.03% ± 0.56%, 66.12% ± 1.77% and 65.76% ± 0.03% at concentrations of 0, 250 and 500 μg/mL respectively. Thus, CARI III-induced growth inhibition of B16F10 cells is correlated with the G0-G1 phase cell cycle arrest.

Eukaryotic cell cycle progression involves sequential activation of CDKs with corresponding regulatory cyclins [8,18]. For instance, the G1-S transition is regulated by complexes of cyclin D and CDK4 or CDK 6 and cyclin E and Cdk2 [8,18]. The cell cycle is precisely controlled by a family of proteins called cyclin-dependent kinases (CDKs), which are positively regulated by cyclins (A, B, D and E) and negatively regulated by cyclin-dependent kinase inhibitors (CDKIs). The G1-phase progression requires the presence of cyclin D-CDK4/6 complexes, and the G1/S phase transition needs cyclin E-CDK2 complexes.

**Figure 8:**
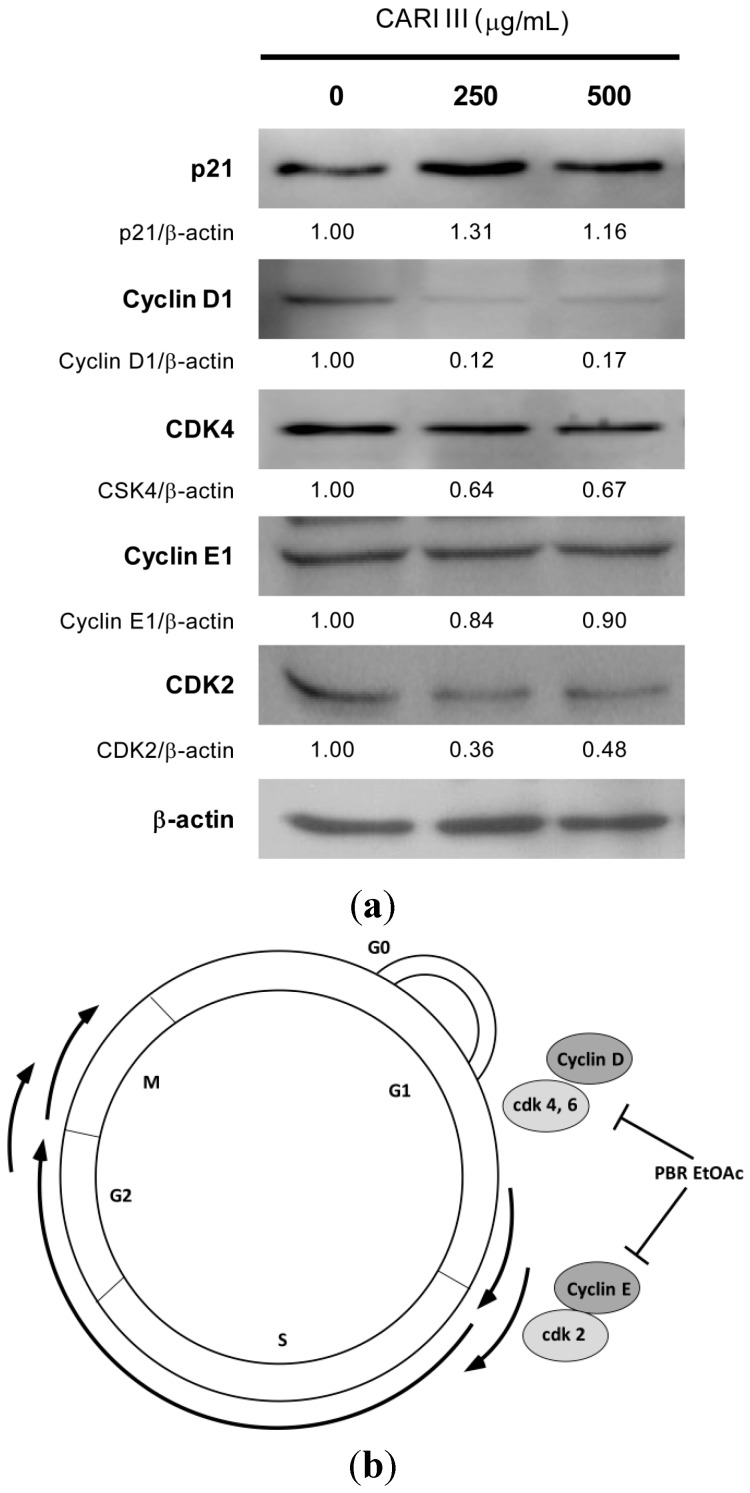
The effect of CARI III extracts on the expression level of cell cycle-related proteins in B16F10 melanoma cells. (**a**) Cells were treated with the indicated concentrations of the CARI III extract for 48 h. Cell lysates were processed for immunoblotting analysis in arbitrary units using anti-p21, cyclin D1, CDK4, cyclin E1, CDK2 and β-actin antibodies. β-actin was used as the internal control. The figures are representative of three independent experiments. The values indicate the density proportion of each protein compared to the control. (**b**) Proposed mechanisms for the effect of CARI III on the cell cycle arrest.

To delineate the molecular mechanism of CARI III-mediated G0/G1 arrest, we determined its effect on the key proteins involved in G0/G1 phase arrest. As can be seen in Figure 8, CARI III treatment caused a rapid and marked decrease in the expression level of cyclin D1, CDK4 and CDK2 proteins in B16F10 cells. The CARI III-treated B16F10 cells showed the decreased level of cyclin D1 and CDK4 protein expression, whereas the level of cyclin E was not altered in this cell line at the 48 h time points (Figure 8). An increase in the expression of p21^Cip1^, a cyclin dependent kinase inhibitor (CDKI), was observed in B16F10 cells by CARI III treatment (Figure 8). p21 is known to negatively regulate the G1 transition. Therefore, the reduction of the G1 phase cell cycle-related proteins of cyclin D1, CDK4 and CDK2 by CARI III might account for the G0/G1 phase arrest (Figure 7). The p21 protein is a well-known CDKI that inhibits the activation of cyclin-CDK2 or -CDK4 complexes. Thus, CARI III functions as a regulator of cell cycle progression at G1. CARI III increased p21 protein expression, which might result in G0/G1 cell cycle arrest.

## 3. Experimental

### 3.1. Preparation of CARI III

CARI III, a dietary supplement, composed of *Phellinus linteus* grown on germinated brown rice (PBR), *Inonotus obliquus* grown on germinated brown rice (IO), *Antrodia camphorata* grown on germinated brown rice (CBR) and *Ganoderma lucidum* (GL), was supplied by CARI, Inc. (Seoul, South Korea). PBR, IO, CBR and GL were ground to a fine powder with a grinder, respectively. The powder was extracted with 80% ethanol (EtOH) for 48 h. The residue was extracted at room temperature and filtered again. The extract was dried by a rotary evaporator under vacuum at 40 °C and stored at −20 °C until use. CARI III, the mixture of PBR, IO, CBR and GL, was dissolved in water and used for the animal experiments.

### 3.2. Experimental Animals

Female C57BL/6 mice at 6 weeks of age were purchased from Orient Bio Inc., Korea, and acclimatized under the controlled conditions for 1 week before the experiment. The animals were fed with commercial rodent feed from Orient Bio Inc., Korea. Food and water were provided *ad libitum* to the animals. All animals were handled following the guidelines of the Institutional Animal Care and Use Committee (IACUC) at Gachon University (Sungnam Gyeonggi-do, Republic of Korea).

### 3.3. Acute Toxicity Test

CARI III was given orally to mice (12 mice/group) at single doses of 1000 mg/kg/day along with the vehicle control. Animals were observed during the first 12 h for any alteration in the symptoms of mobility, posture, the amount of food consumption and for mortality. Mice were weighed daily and observed for fourteen days following treatment.

### 3.4. Tumor Growth Analysis in Vivo

The inhibitory effect of CARI III on melanoma tumor growth was investigated in an animal model. Tumors were induced as previously described [12]. Experiments were performed in six groups: normal control group, saline injection; tumor control group, B16F10 (2 × 10^5^ cells/mouse) injected intraperitoneally (i.p.); CARI III treated group, B16F10 (2 × 10^5^ cells/mouse) injected intraperitoneally; CARI III oral administered group, B16F10 (2 × 10^5^ cells/mouse) injected intraperitoneally; and doxorubicin (Dox) (Sigma, St. Louis, MO, USA) intraperitoneally injected group, B16F10 (2 × 10^5^ cells/mouse) (n = 7 per group). CARI III (300 mg/kg/day) and doxorubicin (1 mg/kg/day) were administered three days before B16F10 melanoma cell transplantation until sacrifice, respectively. Body weight was measured once every three days. The number of surviving animals was examined every day. Tumor was analyzed on Day 14 following B16F10 melanoma cell transplantation. Mice were sacrificed 14 days following cell inoculation. Tumor tissues were collected for further analysis. Tumor was imaged with a digital camera (Power Shot A470; Canon, Tokyo, Japan).

### 3.5. Cell Culture

The B16F10 melanoma cell line was obtained from the American Type Culture Collection (ATCC, Manassas, VA, USA) and cultured in MEM (Invitrogen Co., Carlsbad, CA, USA) containing 10% (v/v) fetal bovine serum, 100 unit/mL penicillin and 100 µg/mL streptomycin in an atmosphere of 95% air and 5% CO_2_ at 37 °C.

### 3.6. Cell Proliferation Assay

Cell proliferation was measured using the Cell Counting Kit-8 (CCK-8) assay (Dojindo Laboratories, Kumamoto, Japan), as described previously [18]. Cells (1 × 10^4^ cells/well) were incubated with the indicated concentrations in the absence or presence of samples for the indicated times. The cell proliferation rate (%) was calculated as the absorbance of sample-treated cells divided by the absorbance of control cells (n = 3). The cell viability of the control group was calculated as 100%. 

### 3.7. Flow Cytometric Analysis of DNA Content for the Cell Cycle

B16F10 melanoma cells (5 × 10^5^ cells/mL) in 6-well plates were incubated in the presence or absence of CARI III extract for the indicated time. After cell harvest, cells were fixed with 70% (v/v) ice-cold ethanol and then incubated with a staining solution containing 0.2% (w/v) NP-40, RNase A (30 μg/mL) (Sigma, St. Louis, MO, USA) and propidium iodide (50 mg/mL) (Sigma, St. Louis, MO, USA) in a phosphate-citrate buffer (pH 7.2). Cellular DNA contents were analyzed by flow cytometry, using a Becton Dickinson laser-based flow cytometer (Becton Dickinson, Franklin Lakes, NJ, USA). At least, 10,000 cells were used for each analysis, and the results were displayed as histograms. Stained cells were run on a FACSCalibur (Becton Dickinson, Franklin Lakes, NJ, USA) with an excitation wavelength of 488 nm and an emission wavelength of 585 nm. Histograms were analyzed using CellQuest software (Becton Dickinson, Franklin Lakes, NY, USA) to determine the cell cycle distribution.

### 3.8. Immunoblot Analysis

Immunoblotting analysis was performed as described previously [19,20]. Total protein (25 μg) was separated by electrophoresis on a 12% SDS-PAGE polyacrylamide gel and then electrophoretically transferred onto polyvinylidene fluoride membranes (PVDF) (Bio-Rad Laboratories, Berkeley, CA, USA). The membranes were incubated with 5% skim milk solution followed by incubation with primary antibody. The membranes were washed in a 1 × TBS-T buffer and incubated with HRP-conjugated secondary antibodies (Santa Cruz, CA, USA, 1:5,000) for 1–2 h. The immunoreactive bands were detected using the enhanced chemiluminescence western blotting detection system (Biosesang, Seoul, Korea).

### 3.9. Statistical Analysis

Data are expressed as means ± standard error of the mean (SEM). Differences between groups were determined by one-way ANOVA followed by Dunnett’s *t*-test. Significant differences were considered from * *p* < 0.05, ** *p* < 0.01,*** *p* < 0.001.

## 4. Conclusions

In conclusion, this is the first investigation demonstrating that CARI III exerted anti-cancer activity that is comparable to Dox against melanoma *in vivo*, as well as *in vitro*. The 300 mg CARI III/kg/day regimen reduced tumor weight, comparable to the doxorubicin (Dox)-treated group, along with increased life span. We also found an inhibitory effect of CARI III on the proliferation of B16F10 melanoma cells. CARI III induced G0/G1 cell cycle arrest by suppressing the expression of cyclin D1 and CDK2 and inducing p21. These data suggest that CARI III might be used as a chemopreventive agent against melanoma.
